# The effect of dimeric bisbenzimidazoles on the activity of DNA repair enzymes TDP1, TDP2, PARP1 and PARP2

**DOI:** 10.18699/vjgb-25-114

**Published:** 2025-12

**Authors:** N.S. Dyrkheeva, I.A. Chernyshova, A.F. Arutyunyan, A.L. Zakharenko, M.M. Kutuzov, K.N. Naumenko, A.S. Venzel, V.A. Ivanisenko, S.M. Deyev, A.L. Zhuze, O.I. Lavrik

**Affiliations:** Institute of Chemical Biology and Fundamental Medicine of the Siberian Branch of the Russian Academy of Sciences, Novosibirsk, Russia; Institute of Chemical Biology and Fundamental Medicine of the Siberian Branch of the Russian Academy of Sciences, Novosibirsk, Russia; The Engelhardt Institute of Molecular Biology of the Russian Academy of Sciences, Moscow, Russia; Institute of Chemical Biology and Fundamental Medicine of the Siberian Branch of the Russian Academy of Sciences, Novosibirsk, Russia; Institute of Chemical Biology and Fundamental Medicine of the Siberian Branch of the Russian Academy of Sciences, Novosibirsk, Russia; Institute of Chemical Biology and Fundamental Medicine of the Siberian Branch of the Russian Academy of Sciences, Novosibirsk, Russia; Institute of Cytology and Genetics of the Siberian Branch of the Russian Academy of Sciences, Novosibirsk, Russia; Institute of Cytology and Genetics of the Siberian Branch of the Russian Academy of Sciences, Novosibirsk, Russia; Shemyakin–Ovchinnikov Institute of Bioorganic Chemistry of the Russian Academy of Sciences, Moscow, Russia; The Engelhardt Institute of Molecular Biology of the Russian Academy of Sciences, Moscow, Russia; Institute of Chemical Biology and Fundamental Medicine of the Siberian Branch of the Russian Academy of Sciences, Novosibirsk, Russia

**Keywords:** tyrosyl-DNA phosphodiesterase 1 (TDP1), TDP1 inhibitor, inhibitory activity, TDP2, PARP1, PARP2, DNA-ligands, bisbenzimidazole derivatives, тирозил-ДНК фосфодиэстераза 1 (TDP1), ингибитор TDP1, ингибирующая активность, TDP2, PARP1, PARP2, ДНК-лиганды, производные бисбензимидазола

## Abstract

Oncological diseases remain a leading cause of pathological mortality worldwide, making the development of anticancer drugs a critical focus in medicinal chemistry. A promising strategy to enhance therapeutic efficacy and reduce chemotherapy-induced toxicity involves the combined inhibition of DNA repair enzymes and topoisomerases. Of particular interest are minor-groove DNA ligands, which exhibit potent inhibition of DNA-dependent enzymes while having low toxicity and mutagenicity. A number of research groups, including ours, are developing inhibitors of DNA repair enzymes that act simultaneously on several targets: tyrosyl-DNA phosphodiesterase 1/2 (TDP1/TDP2), poly(ADP-ribose) polymerase 1 (PARP1)/TDP1, topoisomerase 1 (TOP1)/TDP1. Such bifunctional inhibitors are designed to resolve the problem of tumor cell resistance to known chemotherapy drugs and increase the effectiveness of the latter. In this study, we evaluated the inhibitory activity of 22 minor-groove DNA ligands – bis- and trisbenzimidazoles against four key repair enzymes: TDP1, TDP2, PARP1, and PARP2. Four series of dimeric compounds and their monomeric units were studied. The difference in inhibitory activity of dimeric bisbenzimidazoles depending on the structure of the compound and the enzyme is shown. Our findings reveal distinct structure-activity relationships, with monomeric and dimeric ligands exhibiting potent TDP1 inhibition at micromolar to submicromolar IC50 values (half-maximal inhibitory concentration). Notably, dimeric compounds from the DB2Py(n) and DB3P(n) series demonstrated superior TDP1 inhibition compared to their monomers. In contrast, all tested compounds showed negligible activity against the other three repair enzymes; so, the compounds demonstrate specificity to TDP1. It should be noted that in this work, in the experiments with TDP1 and TDP2, the effect of the tested compounds as narrow-groove ligands binding to DNA was excluded, and their direct effect on the enzyme was investigated. The results of molecular docking suggest the possibility of direct interaction of active compounds with the active center of TDP1. According to the results of modeling, the inhibitors are located in the binding region of the 3’-end of DNA in the active site of TDP1 and could form stable bonds with the catalytically significant TDP1 residues His263 and His493. These interactions probably provide the high inhibitory activity of the compounds observed in biochemical experiments.

## Abstract (ru)

Онкологические заболевания остаются одной из главных причин патологической смертности в мире, что определяет дизайн противораковых препаратов как ключевое направление медицинской химии. Комбинация ингибиторов ферментов репарации ДНК с ингибиторами топоизомераз – перспективный подход для усиления противоракового действия и снижения токсичности химиотерапии. Особый интерес представляют узкобороздочные ДНК-лиганды, способные эффективно ингибировать ДНК-зависимые ферменты, обладая при этом низкой токсичностью и мутагенностью. Ряд исследовательских групп, включая нашу, разрабатывает ингибиторы ферментов репарации ДНК, действующие одновременно на несколько взаимосвязанных мишеней {тирозил-ДНК-фосфодиэстеразы 1/2 (TDP1/TDP2), поли(АДФ-рибоза)полимераза 1 (PARP1)/TDP1, топоизомераза 1 (ТОР1)/TDP1}. Такие бифункциональные ингибиторы призваны решить проблему резистентности опухолевых клеток к известным химиопрепаратам и повысить эффективность последних. В настоящем исследовании представлены данные скрининга ингибирующей активности 22 узкобороздочных лигандов, взаимодействующих с ДНК, – бис- и трисбензимидазолов – в отношении четырех ферментов репарации: TDP1, TDP2, PARP1 и PARP2. Изучены четыре серии димерных соединений и их мономерных единиц. Показана разница в ингибирующей активности димерных бисбензимидазолов в зависимости от структуры соединения и фермента. Мономерные и димерные бисбензимидазолы эффективно ингибируют активность TDP1 в микромолярном и субмикромолярном диапазоне IC50 (концентрация полумаксимального ингибирования). Димерные соединения групп DB2Pу(n) и DB3P(n) проявили более значительную ингибирующую активность в отношении ферментативной реакции с участием TDP1 по сравнению с мономерами, входящими в их состав. Для всех исследованных соединений была показана низкая ингибирующая способность в отношении остальных трех ферментов репарации ДНК, т. е. наблюдается их специфическое воздействие именно на TDP1. Следует отметить, что в данной работе в экспериментах с TDP1 и TDP2 было исключено действие исследуемых соединений как узкобороздочных лигандов, связывающихся с ДНК, и исследовано их непосредственное воздействие на фермент. По результатам молекулярного докинга можно предположить возможность прямого взаимодействия изучаемых соединений с активным центром TDP1. Согласно результатам моделирования, ингибиторы располагаются в области связывания 3’-конца ДНК с активным центром TDP1 и могут образовывать устойчивые связи с каталитически значимыми остатками активного центра His263 и His493. Эти взаимодействия, вероятно, обеспечивают высокую ингибирующую активность соединений, наблюдаемую в биохимических экспериментах.

## Introduction

Nowadays, DNA repair enzymes are actively studied by various researchers to understand the mechanisms of maintaining genetic stability and preventing the development of various diseases. Disruptions in DNA repair systems lead to the accumulation of modified bases, DNA breaks, and other damages, which increase the risk of developing oncological and other diseases. The study of DNA repair system functioning helps to identify the causes of hereditary diseases, neurodegenerative dysfunctions associated with repair defects, and develop new methods for the therapy and prevention of oncological diseases

In recent years, considerable attention has been paid to DNA repair enzymes as targets for drug development. Researchers are actively searching for new compounds that suppress the activity of DNA repair enzymes to enhance the efficacy of anticancer therapy. Inhibition of enzymes involved in repair increases the effectiveness of antitumor therapy, as this leads to cancer cell death due to the accumulation of DNA damage caused by chemotherapy or radiation therapy. Currently, such repair enzymes as tyrosyl-DNA phosphodiesterases 1 and 2 (TDP1 and TDP2) and poly(ADP-ribose) polymerases 1 and 2 (PARP1 and PARP2) are considered promising targets for drug development (Pommier et al., 2014; Curtin, Szabo, 2020; Zakharenko et al., 2023).

TDP1 is a DNA repair enzyme that participates in the removal of covalent adducts of topoisomerase 1 (TOP1) from DNA, catalyzing the hydrolysis of the phosphodiester bond between the Tyr723 residue of TOP1 and the 3′-phosphate group in the single-strand DNA break generated by TOP1. TDP1 is also capable of removing other DNA-protein adducts located at the 3′-end of DNA and various other damage at the 3′-end of DNA (Comeaux, van Waardenburg, 2014; Kawale, Povirk, 2018). TDP2 catalyzes the hydrolysis of covalent adducts between DNA and the Tyr804 residue of the active center of topoisomerase 2 (TOP2) (Pommier et al., 2010). TDP2 removes covalent adducts from DNA located at the 5′- end of DNA through hydrolysis of the 5′-phosphodiester bond, resulting in the formation of DNA with a free 5′-phosphate (Pommier et al., 2014). TDP1 and TDP2 are capable of taking over each other’s functions to some extent, since TDP1 has low activity in the cleavage of 5′-phosphotyrosyl bonds, while TDP2 has low activity in the cleavage of 3′-phosphotyrosyl bonds (Zeng et al., 2012; Pommier et al., 2014).

Today, topoisomerase inhibitors are widely used in clinical practice as anticancer drugs. The most widely used topoisomerase inhibitors are topotecan and irinotecan, which suppress the activity of topoisomerase 1, as well as etoposide, targeting topoisomerase 2 (Pommier et al., 2010). Their mechanism of action consists in the formation of covalent adducts of topoisomerases with DNA, replication arrest, which ultimately leads to the suppression of cell proliferation. Various researchers have expressed the opinion (Pommier et al., 2014; Zakharenko et al., 2015) that the use of TDP1 and TDP2 inhibitors, which enhance the efficacy of topoisomerase inhibitors, may allow reduction of the dose of these rather toxic drugs and, consequently, the toxicity of therapy. Today, the search for TDP1 inhibitors is actively underway (Zakharenko et al., 2023; Zhang M. et al., 2025). As TDP1 inhibitors, derivatives of natural compounds such as usnic acid, berberines, coumarins, nucleosides, and steroids are particularly notable (Zakharenko et al., 2023), which are effective inhibitors of the purified TDP1 enzyme and topotecan sensitizers in experiments conducted on cellular and mouse cancer models (Zakharenko et al., 2023; Kornienko et al., 2024). Among TDP2 inhibitors, deazaflavins are worth noting, being among the most active inhibitors found to date for this enzyme (Marchand et al., 2016).

The enzymes PARP1 and PARP2 are key regulators of DNA repair and other cellular processes. These enzymes catalyze the DNA-dependent synthesis of the branched polymer poly(ADP-ribose) (PAR) and subsequent ADP-ribosylation of proteins. ADP-ribosylation of proteins is a post-translational modification that is induced in response to DNA damage. PARP1 participates in various DNA repair pathways (Ray Chaudhuri, Nussenzweig, 2017; Lavrik, 2020). PARP2 is also a DNA-dependent PARylation agent and can partially replace PARP1 (Lavrik, 2020; Szanto et al., 2024); therefore, the search for PARP1 and PARP2 inhibitors is an urgent task of modern medicinal chemistry. In clinical practice, such PARP1 and PARP2 inhibitors as olaparib, rucaparib, niraparib, veliparib, and talazoparib are currently approved for use in the treatment of ovarian, fallopian tube, breast, and peritoneal cancer (Kim D.-S. et al., 2021). The inhibitors used today work on the principle of synthetic lethality to destroy cancer cells with defects in the homologous recombination system (for example, with BRCA1/2 mutations), converting single-strand DNA breaks into double-strand breaks that cannot be effectively repaired, leading to cancer cell death. The active sites of PARP1 and PARP2 are very similar (Schreiber et al., 2006; Hoch, Polo, 2019); therefore, the currently known inhibitors most often act on both enzymes, as well as on other enzymes of the PARP family, due to the similarity of their active center that binds nicotinamide adenine dinucleotide (NAD+) and initiates the synthesis of poly(ADP-ribose), therefore the search for selective inhibitors of each of these enzymes is actively conducted (Johannes et al., 2024). PARP inhibitors approved for clinical use are quite toxic and cause severe side effects, so the search for new inhibitors actively continues (Murai et al., 2014; Kim D.-S. et al., 2021; Johannes et al., 2024).

Small-molecule DNA-binding agents are an extremely promising class of compounds for the search of new inhibitors of repair enzymes. Of particular interest are minor-groove DNA ligands capable of inhibiting DNA-dependent enzymes, while not possessing high toxicity and mutagenicity, and being well soluble in water. Such DNA ligands have a low level of DNA geometry alteration and absence of covalent crosslink formation when forming a complex with DNA (Arutyunyan et al., 2023a).

Our research group has significant experience both in experimental investigation of potential inhibitors at the level of individual protein targets, cells, and animal models (Zakharenko et al., 2023), and in the application of molecular docking and modeling methods to study the mechanisms of interaction of small molecules with target proteins. Effective TDP1 inhibitors have been found that inhibit the recombinant TDP1 enzyme in the submicromolar concentration range. The lead compounds were topotecan sensitizers in experiments conducted on cell cultures and mouse tumor models (Zakharenko et al., 2023; Kornienko et al., 2024). We have developed and investigated inhibitors of PARP1, PARP2, and PARP3 based on conjugates of ADP and morpholino nucleosides using structural modeling of the active sites of these enzymes (Sherstyuk et al., 2019; Chernyshova et al., 2024).

**Fig. 1.**
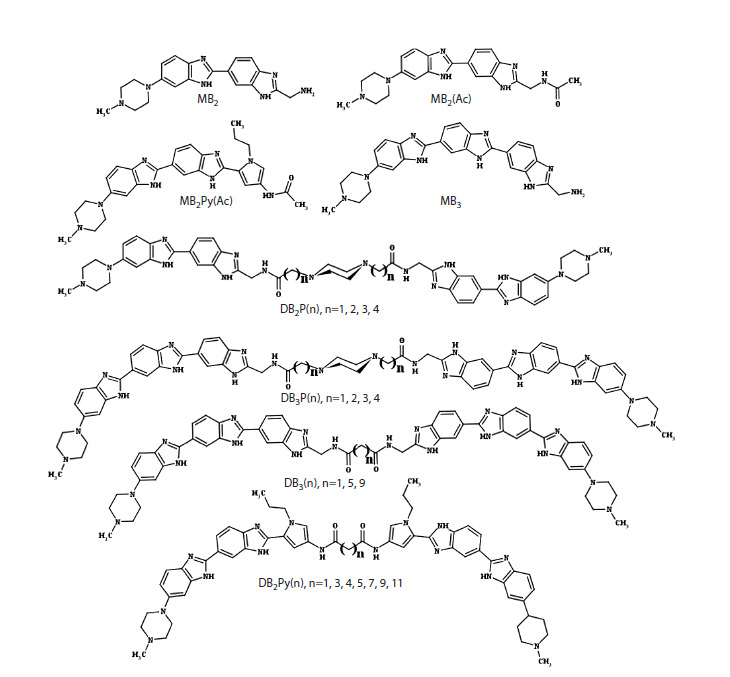
Structures of bisbenzimidazole derivatives studied in this work.

This work presents screening data of twenty-two minorgroove ligands as inhibitors of TDP1, TDP2, PARP1, and PARP2. The studied compounds are bis- and trisbenzimidazole derivatives. Four monomeric compounds – MB2, MB2(Ac), MB2Py(Ac), MB3 – as well as four series of dimeric derivatives were investigated. The dimeric derivatives were obtained by condensation of monomeric subunits with dicarboxylic acids DB2P(n), DB2Py(n), and DB3P(n), where (n) is the number of methylene units in the linker (Fig. 1).

It was shown that the activity of the compounds varies depending on their structure and the type of enzymatic target. The studied compounds exhibited pronounced inhibitory activity against TDP1, and the observed correlation indicates an increase in inhibitor activity upon introduction of addi- tional binding blocks into its structure, such as a pyrrolecarboxamide fragment for the DB2Py(n) series, or when using a combination of three benzimidazole blocks in the monomeric subunit. Despite the fact that extremely high IC50 values were observed for the DB3(n) series, this phenomenon can be explained by the high propensity of members of this series of compounds to aggregation, since the introduction of a piperazine fragment into the linker in the DB3P(n) series led to the obtaining of inhibitors with the lowest IC50 values, which indirectly confirms our assumption. In order to elucidate the possible mechanism of their inhibitory action for this enzyme, molecular docking was performed, the results of which suggest the presence of direct interaction between the active compounds and the TDP1 enzyme. According to the constructed binding model, the inhibitors are located in the region of the DNA-binding pocket of TDP1 and are capable of forming stable contacts with the catalytically important amino acid residues His263 and His493. The efficacy of these compounds as TDP1 inhibitors was confirmed by experimental data. The results of the work can be used for the rational design of new, even more effective TDP1 inhibitors.

## Materials and methods

Materials and reagents. The studied compounds were synthesized at the Engelhardt Institute of Molecular Biology in the Laboratory of DNA-Protein Interactions according to previously developed methods (Ivanov et al., 2015; Arutyunyan et al., 2023a, b; Susova et al., 2024). The list of IUPAC names of the compounds is provided in the Supplementary Materials1


Supplementary Materials are available in the online version of the paper: https://vavilov.elpub.ru/jour/manager/files/Suppl_Dyrkheeva_Engl_29_7.pdf


Recombinant human proteins tyrosyl-DNA phosphodiesterase 1 (TDP1) and tyrosyl-DNA phosphodiesterase 2 (TDP2) were expressed in the E. coli system, poly(ADP-ribose) polymerase 1 (PARP1) and poly(ADP-ribose) polymerase 2 (PARP2) were expressed in insect cells using a baculovirus expression system and purified as described in (Sukhanova et al., 2004; Sherstyuk et al., 2019; Dyrkheeva et al., 2020, 2021).

The oligonucleotide 5′-FAM-AAC GTC AGG GTC TTC C-BHQ1-3′ was synthesized at the Laboratory of Nucleic Acid Chemistry, Institute of Chemical Biology and Fundamental Medicine (Novosibirsk, Russia), according to (Zakharenko et al., 2015).

Determination of TDP1 activity. The reaction mixture (200 μl) for real-time fluorescent detection of TDP1 enzyme activity (Zakharenko et al., 2015) contained TDP1 reaction buffer (50 mM Tris-HCl, pH 8.0, 50 mM NaCl, and 7 mM β-mercaptoethanol), 50 nM oligonucleotide 5′-FAM-AAC GTC AGG GTC TTC C-BHQ1-3′, the test compound at various concentrations, and TDP1 at a final concentration of 1.5 nM. The reaction mixtures were incubated at a constant temperature of 26 °C in a POLARstar OPTIMA microplate fluorometer (BMG LABTECH, GmbH, Ortenberg, Germany). Fluorescence intensity (Ex485/Em520 nm) was measured every minute for 10 min. Mean values of half-maximal inhibitory concentration (IC50 – the concentration of the compound that inhibited 50 % of enzyme activity compared to the untreated control well containing only enzyme and substrate) were determined using a dose-response curve of the fluorescence signal level versus inhibitor concentration and calculated using MARS Data Analysis 2.0 (BMG LABTECH). Kinetic curves were obtained in at least three independent experiments and statistically processed in OriginPro 8.6.0 (OriginLab, Northampton, Massachusetts, USA).

Determination of TDP2 activity. For determination of TDP2 enzyme activity, an oligonucleotide 5′-tyrosine-AAC GTC AGG GTC TTC C-FAM-3′ containing a 6-FAM label at the 3′-end and an L-tyrosine residue attached via the phenolic OH group to the 5′-terminal phosphate was used as substrate, synthesized at the Russian-French-Japanese Laboratory of Bionanotechnology of Novosibirsk State University as described in (Dyrkheeva et al., 2021). The substrate at a concentration of 100 nM was incubated with TDP2 at a concentration of 200 nM in the absence or presence of inhibitor (500 μM) for 10 min at 37 °C in buffer containing 50 mM Tris-HCl, pH 8.0, 50 mM NaCl, 7 mM β-mercaptoethanol (Dyrkheeva et al., 2021). The reaction was stopped by addition of PAGE loading buffer (TBE, 10 % formamide, 7 M urea, 20 mM EDTA, 0.1 % xylene cyanol, and 0.1 % bromophenol blue). The samples were then heated at 90 °C for 5 min. The enzymatic reaction products were separated by electrophoresis in 20 % denaturing PAGE with 7 M urea at an acrylamide to bisacrylamide ratio of 19:1. A high-resolution Typhoon FLA 9500 laser scanner (GE Healthcare, Chicago, Illinois, USA) was used for gel scanning and visualization, and the data were analyzed using QuantityOne 4.6.7 software (Bio- Rad Laboratories, Inc., Hercules, California, USA). At least three independent experiments were performed, and statistical processing was carried out using OriginPro 8.6.0 (OriginLab, Northampton, Massachusetts, USA).

Determination of PARP1 and PARP2 activity. For determination of PARP1 and PARP2 enzyme activity in the presence and absence of test compounds, radiolabeled [32P]-NAD⁺ was synthesized from α-[32P]-ATP according to the protocol (Sherstyuk et al., 2019). The auto-poly(ADPribosyl) ation reaction was performed in buffer for PARP1: 50 mM Tris-HCl, pH 8.0, 10 mM MgCl2, 150 mM NaCl, and 7 mM β-mercaptoethanol, as well as 2 A260 units/ml activated DNA, 0.3 mM [32P]-NAD⁺ at 37 °C. The reaction was initiated by addition of PARP1 to 200 nM and carried out for 2 min

The buffer for PARP2 contained: 50 mM Tris-HCl, pH 8.0, 3 mM spermine, 150 mM NaCl, and 7 mM β-mercaptoethanol, 2 A260 units/ml activated DNA, 0.6 mM [32P]-NAD⁺ at 37 °C. The reaction was initiated by addition of PARP2 to 600 nM, and the reaction mixtures were incubated for 5 min. The reaction was stopped by placing 5 μl aliquots on Whatman 1 paper filters impregnated with 5 % trichloroacetic acid (TCA). The filters were washed with 5 % TCA four times and air-dried after removal of TCA with 90 % ethanol. The incorporation of the radioactive label into the reaction product was calculated using a Typhoon FLA 9500 scanner (GE Healthcare, Chicago, Illinois, USA). At least three independent experiments were performed.

Molecular modeling. To evaluate the interaction of the studied compounds with the TDP1 enzyme, we performed molecular docking followed by analysis of the resulting complexes. The study included preparation of protein and ligand structures, molecular docking, energy minimization of compounds in the binding site, and assessment of inhibitor affinity using the Vinardo, X-Score, and REF2015 scoring functions.

The crystal structure of TDP1 (PDB ID: 8V0B) was used as the target protein structure. Missing loops in the model were reconstructed based on AlphaFold2 prediction (Jumper et al., 2021) performed in ColabFold (Mirdita et al., 2022) without using multiple sequence alignment (MSA) and using 8V0B as a template.

Hydrogen atoms were then added to the resulting model and charges were calculated using the DockPrep utility in UCSF Chimera (Pettersen et al., 2004). The inhibitor structures were prepared in OpenBabel (O’Boyle et al., 2011): hydrogens were added, partial charges were calculated, and geometry minimization was performed

Molecular docking was performed using the UCSF DOCK 6.11 software package (Allen et al., 2015). Fullatom flexible docking over the entire protein surface was used. At the first stage of docking, the core fragments of the inhibitors (MB2(Ac), MB2Py(Ac)) were positioned, after which full-length molecules were docked with subsequent minimization of their energy in the binding site. Up to nine best conformations by GridScore were requested for each compound. From the nine conformations obtained for each ligand, the structure with the minimum RMSD relative to the optimal conformation of the core fragment was selected. In cases where DOCK6 returned fewer than nine unique conformations (due to clustering, energy filtering, or failure to generate additional conformers), selection was performed from all available conformations (Table S1).

Final assessment of the inhibitors’ binding ability to the protein was performed using several independent scoring functions: ContinuousScore from DOCK 6, Vinardo (Quiroga, Villarreal, 2016), X-Score (Wang R. et al., 2002), and REF2015 in the PyRosetta4 environment (Chaudhury et al., 2010; Alford et al., 2017) according to the protocol of Moretti et al. (2016). ContinuousScore is a scoring function in DOCK 6 that accounts for van der Waals interactions, electrostatic interactions, internal ligand energy, and penalties for steric clashes through direct calculation of interatomic distances. Vinardo is a scoring function for docking that accounts for the contribution of hydrogen bonds, hydrophobic and van der Waals interactions, as well as corrections for non-optimal ligand positioning. The X-Score scoring function consists of three components: HPScore, HMScore, and HSScore, based on different empirical principles for assessing ligand-protein affinity. In this study, the averaged X-Score was used, reflecting the influence of hydrophobic, polar, and electrostatic contacts. The full-atom REF2015 scoring function implemented in PyRosetta includes contributions from van der Waals, electrostatic, hydrogen bonding, solvation, and additional atom pair interactions and allows correct ranking of inhibitor positions close in energy.

To validate the molecular docking results and assess the stability of the predicted complex over time, molecular dynamics simulation of the TDP1 complex with the lead compound DB2Py(1), which had shown the best inhibitory activity, was performed. The simulation was carried out using the OpenMM 8 package (Eastman et al., 2017). A detailed protocol of the molecular dynamics simulation is presented in the Supplementary Materials.

## Results

In this work, the ability of four series of small-molecule dimeric DNA ligands DB2P(n), DB2Py(n), DB3(n), DB3P(n) as well as their monomeric units MB2, MB2(Ac), MB2Py(Ac), and MB3 (Fig. 1) to inhibit the activity of recombinant DNA repair enzymes TDP1 and TDP2, PARP1 and PARP2 was studied for the first time (see the Table).

**Table 1.**
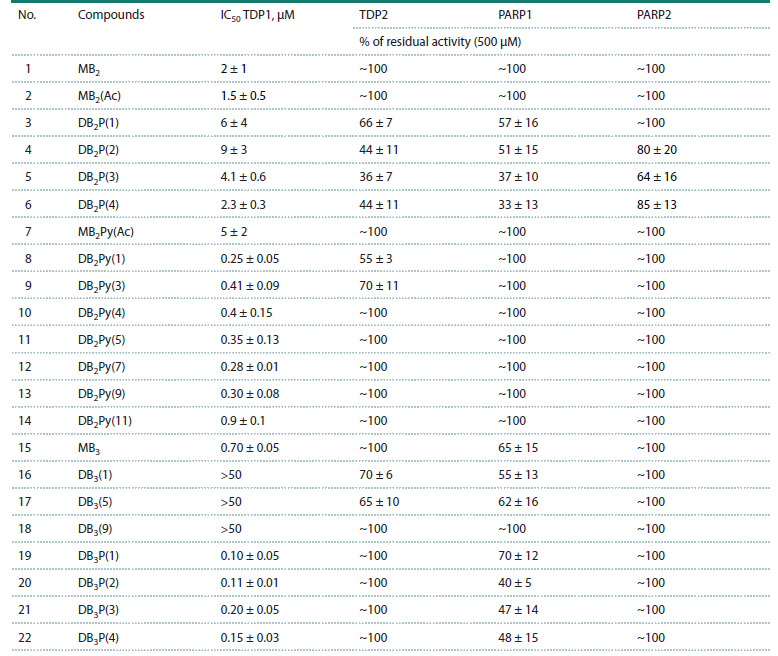
Inhibitory activity of test compounds against TDP1, TDP2, PARP1, and PARP2 Note. For IC50 values and percentage of residual enzyme activity in the presence of inhibitor, the Table shows mean values ± standard deviation (at least three replicates).

The first group of studied compounds represents dimeric derivatives of the monomeric bisbenzimidazole ligand MB2, a derivative of the widely studied minor-groove DNA ligand Hoechst 33258, in which the hydroxyphenyl group is replaced by a more hydrophilic aminomethylene fragment – DB2P(n). As a linker for compounds of this group, 1,4-piperazinedialkyldicarboxylic acids containing a methylene, ethylene, propylene, or butylene spacer were used (Fig. 1). This series was also supplemented with the monomeric derivative MB2(Ac), acylated at the aminomethylene fragment, which structurally brings this compound, compared to MB2, closer to half of the dimeric compound DB2P(n) and makes it a more appropriate reference for comparison. The DB2P(n) series differs from other ligand series by the presence of a positively charged 1,4-piperazine introduced into the linker, which improves ligand solubility and may increase ligand affinity for the enzyme.

The next group of compounds are derivatives of the monomeric trisbenzimidazole compound MB3, which can be considered as a derivative of MB2 containing one additional benzimidazole fragment, which increases the number of potentially possible hydrogen bonds in the inhibitor-TDP1 complex. Dimeric derivatives of MB3 are represented by two series of compounds – DB3P(n), also dimerized with 1,4-piperazinedialkyldicarboxylic acids, and DB3(n), where n-alkyldicarboxylic acids are used as linkers. The DB3(n) and DB3P(n) series are characterized by the presence of trisbenzimidazoles in the structure, and DB3P(n), also by the presence of 1,4-piperazine in the linker.

The third group of compounds includes derivatives of the monomeric compound MB2Py(Ac), which is an isosteric analog of MB3, due to the fact that the pyrrolecarboxamide fragment contained in its structure can act as a hydrogen atom donor at the carboxamide nitrogen for hydrogen bond formation, in a position analogous to benzimidazole. Dimeric derivatives are represented by the DB2Py(n) series containing n-alkyldicarboxylic acids as a linker. This series is represented by a set of compounds containing 1, 3, 4, 5, 7, 9, and 11 methylene units, which allowed for a more accurate assessment of the dependence of the inhibitory activity of compounds on spacer length. The DB2Py(n) series differs from the DB3(n) series by the presence, in addition to the bisbenzimidazole structure, of a pyrrolecarboxamide structure, which is a fragment of the AT-specific antibiotic netropsin.Using the real-time fluorescence analysis method, halfmaximal inhibitory concentration (IC50) values of the studied compounds (see the Table) were obtained in the reaction of BHQ1 cleavage from the 3′-end of the oligonucleotide by TDP1, which led to an increase in FAM fluorescence at the 5′-end of the chain (Zakharenko et al., 2015). It should also be noted that a single-stranded oligonucleotide was used as substrate to exclude the binding of dimeric bisbenzimidazoles as minor-groove ligands to the DNA substrate and direct their action toward the enzymatic target.

From the data obtained for the monomeric compounds MB2 and MB2(Ac) and their dimeric derivatives DB2P(n), at n = 1, 2, 3, 4, the IC50 values were in the micromolar range, and dimerization did not lead to an increase in the inhibitory activity of the studied compounds. At the same time, for dimers of the monomeric MB2Py(Ac), which has an IC50 value of 5 ± 2 μM, the half-inhibitory concentration parameter value decreased significantly, ranging from 0.25 to 0.90 μM. Similarly, the transition from monomeric MB3 to the dimeric DB3P(n) series led to an increase in the inhibitory activity of the compounds, although not as pronounced; however, dimeric derivatives of MB3 that do not contain a piperazine fragment in the linker – DB3(n) compounds – showed the lowest level of activity among all the inhibitors tested in this work. The fact that the IC50 values for these compounds (see the Table) deviate so strongly from the overall data set is most likely due to the fact that DB3(n) compounds possess an extended and planar geometry, as well as a rigid linker, which prevents optimal positioning of compounds of this type in the enzyme active site (Fig. 1).

Thus, according to the experimental data, all compounds studied in this work, except for the DB3(n) group, effectively inhibit TDP1 activity at micromolar and submicromolar concentrations. A structure-activity correlation is observed, consisting of a decrease in concentration to achieve the halfmaximal inhibition effect with an increase in the number of blocks containing hydrogen bond donors in the compound. In particular, dimerization is one of the simple approaches to increasing such structures in one molecule, which leads to a nonlinear increase in the binding constant (Neudachina, Lakiza, 2014). A decrease in IC50 is also observed upon introduction of a piperazine fragment into the linker structure, which may be due to an increase in the hydrophilicity of the molecules. The results obtained allowed us to establish a structure-activity correlation, as well as to assess the contribution of dimerization to the increase of the inhibitory capacity of the studied compounds.

To study the effect of the studied compounds on TDP2 activity, we tested the ability of this enzyme to remove the tyrosine residue from the 5′-end of the oligonucleotide substrate in the absence and presence of inhibitors, as described in (Dyrkheeva et al., 2021). All compounds of the DB2P(n) group, as well as DB2Py(n), at n = 1, 3 and DB3(n), at n = 1, 5 at a concentration of 500 μM inhibited enzyme activity by approximately 50 %, while all other compounds showed no inhibitory activity (see the Table). Thus, all tested compounds showed a significantly lower propensity to inhibit TDP2 compared to TDP1. Interestingly, the DB2P(n) group inhibited TDP1 less effectively and TDP2 more effectively than compounds of other groups

The next step of our work was to test the ability of the studied compounds to inhibit PARP1 and PARP2, that is, their enzymatic activity in the poly(ADP-ribose) (PAR) synthesis reaction, at a rather high concentration range of compounds. All studied compounds showed low efficiency in inhibiting these two enzymes. The most active compounds were those of the DB2P(n) group, representatives of which with n = 2, 3, 4 reduced the activity of PARP1 and PARP2 at a concentration of 500 μM. Inhibitory action was also observed for compounds of the DB3(n) and DB3P(n) series at a concentration of 500 μM, while these compounds exhibited inhibitory activity only in the PAR synthesis reaction catalyzed by PARP1, but not PARP2 (see the Table).

Since, according to the experimental data, all studied compounds, with the exception of the DB3(n) group, effectively inhibit TDP1 activity, we further performed an in silico evaluation of the ability of compounds of the DB2P(n) and DB2Py(n) groups to bind to the TDP1 enzyme in order to elucidate the possible molecular mechanism of their inhibitory action. For this purpose, full-atom flexible molecular docking over the entire surface of the TDP1 protein (PDB ID: 8V0B) was performed for DB2P(n) and DB2Py(n) compounds.

**Fig. 2.**
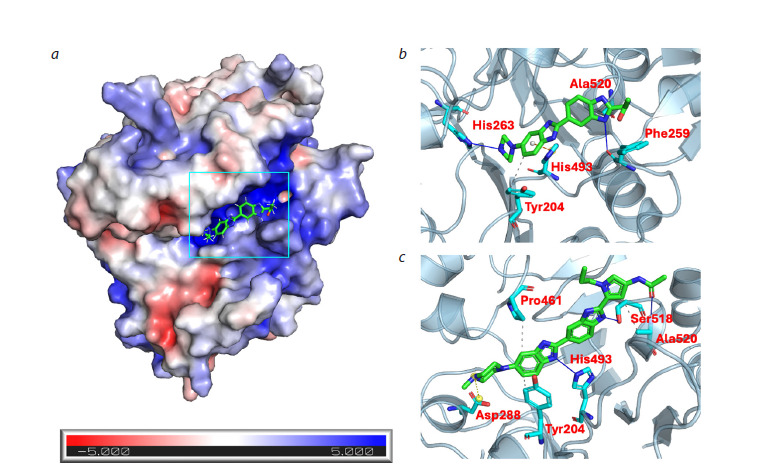
a, Structure of TDP1 (PDB ID: 8V0B) with inhibitor MB2(Ac) located in the positively charged region of the TDP1 active site. The protein surface is colored according to the electrostatic potential distribution calculated using APBS (Jurrus et al., 2018). The DNA-binding region of TDP1 is highlighted by a rectangular frame. Below is a scale of TDP1 surface electrostatic potential values (in units of kT/e, where kT/e ≈ 25.7 mV at 298 K). Color scale: red indicates negative potential (−5 kT/e), white indicates neutral (0 kT/e), blue indicates positive potential (+5 kT/e). b, c, Predicted conformations of inhibitors MB2(Ac) and MB2Py(Ac) (green) in complex with TDP1 with contacting residues (cyan).

According to molecular modeling data, compound MB2(Ac) (Fig. 2b), which is the monomeric unit for dimeric derivatives DB2P(n), may form a hydrogen bond with His263 and a π-cation interaction with His493, which could potentially lead to blocking of the TDP1 catalytic act. In addition to interactions with catalytically active residues, MB2(Ac) may form hydrophobic contacts with Tyr204 and Ala520, as well as a hydrogen bond with Phe259, which could enhance the inhibitory action of this compound. In contrast to MB2(Ac), compound MB2Py(Ac) (Fig. 2c) appears to interact with only one catalytic residue – His493 – through hydrogen bond formation. Such a difference in interactions could be the reason for the higher inhibitory activity of MB2(Ac) compared to MB2Py(Ac), which is consistent with experimental data (see the Table).

Analysis of interactions using PLIP (Protein–Ligand Interaction Profiler) (Salentin et al., 2015) for predicted TDP1 complexes with dimeric compounds of the DB2Py(n) group (Fig. S2) showed that these compounds form a greater number of protein-ligand contacts (hydrogen bonds and hydrophobic interactions) compared to the MB2Py(Ac) monomer. In particular, compound DB2Py(1) forms hydrogen bonds with Ser400 and Ser403, as well as hydrophobic interactions with Pro463 – the residues of these amino acids are located in the ligand binding site with the TDP1 active center, which likely contributes to stabilization of the interacting dimer fragment in the enzyme active site. The data obtained from docking analysis, characterizing the larger contact surface area of dimeric DB2Py(n) compounds with TDP1 compared to the MB2Py(Ac) monomer, correlate with the decrease in IC50 values for dimers, which indicates an increase in the affinity of these compounds for the enzyme active site (see the Table). According to the data obtained, hydrophobic interactions with Pro461 and/or Tyr204 residues localized in the TDP1 active site may also contribute to increasing the inhibitory activity of DB2Py(n) group compounds.

Analysis of interactions of compounds from the DB2P(n) group with TDP1 showed that analogous amino acid residues participate in complex formation, with the exception of Tyr204, with which DB2P(n) compounds, unlike DB2Py(n), apparently do not interact (Fig. S3). In addition, possible differences in the nature of interactions with the same amino acids were noted. For example, for the Lys519 residue in the case of DB2P(n) compounds, formation of hydrogen bonds with nitrogen atoms of the piperazine fragment through the N1 atom of the side chain can be assumed. At the same time, two types of interactions with Lys519 are predicted in DB2Py(n) compounds: a hydrogen bond between the backbone nitrogen atom of Lys519 and the oxygen atom in the pyrrolecarboxamide group (in DB2Py(1), DB2Py(4), DB2Py(7), DB2Py(9)), as well as a π-cation interaction between pyrrole and the Lys519 side chain (in DB2Py(3) and DB2Py(5)) (Fig. S2).

For compound DB2Py(1), which demonstrated the highest inhibitory activity (lowest IC50 value) among the studied derivatives, additional molecular dynamics modeling in the predicted complex with TDP1 was performed. Analysis of the MD trajectory showed that the TDP1-DB2Py(1) complex maintains stability throughout the simulation time. RMSD values of the ligand were in the range of 1.5–3.0 Å (Fig. S4), which indicates stable binding of DB2Py(1) in the protein active site without signs of dissociation or significant conformational rearrangements. The data obtained confirm the strength of the formed complex and are consistent with the high biological activity of this compound.

It should be noted that our analysis of molecular contacts, as well as the scoring function values obtained according to molecular docking results, indicate the ability of compounds of both analyzed groups – DB2P(n) with an aliphatic linker and DB2Py(n) with a piperazine fragment in the linker – to form a stable complex with TDP1. Nevertheless, experimental data show differences in their inhibitory activity: compounds with an aliphatic linker demonstrate higher inhibition efficiency compared to compounds containing a piperazine ring. This difference cannot be fully explained based on contact analysis, which suggests a possible difference in the conformational mobility of these groups of compounds. In particular, the inclusion of a piperazine fragment in the central part of the linker apparently restricts its flexibility, which affects the dynamics of inhibitor interaction with the active site, prevents optimal positioning of the inhibitor in the enzyme active site and, consequently, reduces its inhibitory activity.

## Discussion

TDP1 plays a key role in eliminating DNA damage located at the 3′-end of DNA, stabilized by anticancer drugs used in clinical practice, such as topotecan and irinotecan, which are derivatives of the natural compound camptothecin (Comeaux, van Waardenburg, 2014; Kawale, Povirk, 2018). Consequently, TDP1 activity may be a possible cause of tumor resistance to TOP1 inhibitors used in the clinic. Currently, searches for combined TOP1 and TDP1 inhibitors are actively underway (Conda-Sheridan et al., 2013; Nguyen et al., 2015; Zhang X.-R. et al., 2018; Hu et al., 2021;Yang et al., 2023).

Furthermore, since it is known that the activities of TDP1 and TDP2 overlap, albeit to a minor extent (Pommier et al., 2014), the ability of these enzymes to perform each other’s functions makes the combined use of inhibitors of these two enzymes or the creation of agents capable of simultaneously inhibiting both TDP1 and TDP2 quite promising. Simultaneous suppression of the activity of these two enzymes can be used to enhance the efficacy of a large set of clinically important anticancer drugs, TOP1 and TOP2 inhibitors. Triple TOP1/TDP1/TDP2 inhibitors have also been discovered, which exhibit moderate activity against TDP1 and weak activity against TDP2 (Wang P. et al., 2017). The most effective TDP2 inhibitors to date are deazaflavins, which exhibit synergy with etoposide in vitro at non-toxic concentrations (Marchand et al., 2016), and some effective TDP2 inhibitors from other compound classes have also been found (Yang et al., 2021; Zhang Y. et al., 2021).

It is known that the N-terminal domain of TDP1 directly binds to the C-terminal domain of PARP1, and TDP1 undergoes PARylation by PARP1 in order to be recruited to the TOP1-DNA adduct (Das et al., 2014; Lebedeva et al., 2015). PARylation of TDP1 stimulates its recruitment to sites with damaged DNA without affecting the catalytic activity of this enzyme (Chowdhuri, Das, 2021). It has also been shown that PARP1 can interact with TDP1, forming protein-protein contacts (Moor et al., 2015). It was established that the combination of TDP1 knockdown and inhibition of PARP1 activity with rucaparib reduces cell proliferation more significantly than these methods of enzyme function suppression separately (Fam et al., 2013). Therefore, there is a suggestion in the literature that the anticancer effect of TOP1 inhibitors can be significantly enhanced by simultaneous inhibition of PARP1 and TDP1 (Smith et al., 2005; Alagoz et al., 2014; Das et al., 2014; Murai et al., 2014; Elsayed et al., 2016; Matsuno et al., 2018; Jing et al., 2020; Kim J.W. et al., 2020; Chowdhuri, Das, 2021; Flörkemeier et al., 2022). The interaction between PARP1 and TDP1 enzymes has been demonstrated in a number of publications (Das et al., 2014; Moor et al., 2015), which makes the search for dual TDP1 and PARP1 inhibitors relevant.

Previously, we discovered dual TDP1 and TDP2 inhibitors, as well as triple TDP1, TDP2, and PARP1 inhibitors (Dyrkheeva et al., 2021) – usnic acid thioethers that weakly inhibit TDP2 and PARP1; therefore, the search for new compounds capable of acting on two or three functionally interacting targets simultaneously is relevant. In this work, the ability of a series of minor-groove DNA ligands to inhibit TDP1, TDP2, PARP1, and PARP2 enzymes was tested. Effective inhibitors acting on all four enzymes simultaneously were not found, but it was shown that these compounds inhibit TDP1. The DNA ligands studied in this work are capable of inhibiting DNA-dependent enzymes through binding to double-stranded DNA. However, in the present work we showed that they are capable of selectively inhibiting TDP1, since the experiments were conducted in the absence of double-stranded DNA as an alternative target.

The results of molecular docking and analysis of intermolecular interactions suggest that most of the studied compounds of the DB2P(n) and DB2Py(n) groups may possess high affinity for the TDP1 enzyme and form stable complexes with its catalytic center. Interactions with key catalytic residues of the TDP1 protein active site were predicted for all compounds

## Conclusion

In this work, a study of the effect of dimeric bis- & tris-benzimidazoles on the activity of DNA repair enzymes – TDP1, TDP2, PARP1, and PARP2 – was conducted. The main results showed that all studied inhibitors, except compounds of the DB3(n) series, effectively inhibit TDP1. The most active were compounds DB2Py(n) and DB3P(n), capable of inhibiting TDP1 in the submicromolar concentration range. The studied compounds demonstrate high selectivity, with minimal effect on the activity of other tested enzymes.

Based on the results of molecular docking, it is proposed that the studied active inhibitors are localized in the region of the DNA-binding pocket of TDP1 and may form stable interactions with the catalytically important residues His263 and His493. These interactions likely underlie the observed high inhibitory activity.

An important result is also the establishment of the structure- activity relationship. Dimerization had a mixed effect on the inhibitory effect: compounds of the DB2Py(n) and DB3P(n) series were significantly (by an order of magnitude) more active than the corresponding monomers; in the DB2P(n) series, the inhibitory activity was influenced not only by dimerization, but also by linker length and the introduction of 1,4-piperazine bearing two positive charges into the linker. The DB3(n) series was inactive, unlike the monomer. Introduction of the piperazine fragment into the linker in the DB3P(n) series led to pronounced inhibitory activity compared to DB3(n) without such a fragment. We propose that the enhancement of the inhibitory effect is related to the introduction of two positive charges into the linker and to the increase in the number of possible contacts of ligands with the enzyme active site

Overall, based on the results of this work, new strategies for the development of cancer therapy may be proposed. The obtained data also highlight the potential of dimeric bis- & tris-benzimidazoles as safe and effective tools for targeted regulation of DNA repair enzymes

## Conflict of interest

The authors declare no conflict of interest.

## References

Alagoz M., Wells O.S., El-Khamisy S.F. TDP1 deficiency sensitizes
human cells to base damage via distinct topoisomerase I and PARP
mechanisms with potential applications for cancer therapy. Nucleic
Acids Res. 2014;42(5):3089-3103. doi 10.1093/nar/gkt1260

Alford R.F., Leaver-Fay A., Jeliazkov J.R., O’Meara M.J., DiMaio F.P.,
Park H., Shapovalov M.V., … Das R., Baker D., Kuhlman B., Kortemme
T., Gray J.J. The Rosetta all-atom energy function for macromolecular
modeling and design. J Chem Theory Comput. 2017;
13(6):3031-3048. doi 10.1021/acs.jctc.7b00125

Allen W.J., Balius T.E., Mukherjee S., Brozell S.R., Moustakas D.T.,
Lang P.T., Case D.A., Kuntz I.D., Rizzo R.C. DOCK 6: impact of
new features and current docking performance. J Comput Chem.
2015;36(15):1132-1156. doi 10.1002/jcc.23905

Arutyunyan A.F., Kostyukov A.A., Korolev S.P., Gottikh M.B.,
Kaluzhny
D.N., Susova O.Yu., Zhuze A.L. DNA sequence-specific
ligands. 19. Synthesis, spectral properties, virological and biochemical
studies of DB3(n) fluorescent dimeric trisbenzimidazoles. Mol
Biol. 2023a;57(3):512-521. doi 10.1134/s0026893323030020

Arutyunyan A.F., Kostyukov A.A., Lushpa V.A., Mineev K.S., Korolev
S.P., Gottikh M.B., Klimova R.R., Kushch A.A., Kalabina K.V.,
Susova O.Yu., Zhuze A.L. DNA sequence-specific ligands. XX. Synthesis,
spectral properties, virological and biochemical studies of
fluorescent dimeric trisbenzimidazoles DB3P(n). Med Chem Res.
2023b;32(3):587-599. doi 10.1007/s00044-023-03017-x

Chaudhury S., Lyskov S., Gray J.J. PyRosetta: a script-based interface
for implementing molecular modeling algorithms using Rosetta.
Bioinformatics. 2010;26(5):689-691. doi 10.1093/bioinformatics/
btq007

Chernyshova I., Vasil’eva I., Moor N., Ivanisenko N., Kutuzov M.,
Abramova T., Zakharenko A., Lavrik O. Aminomethylmorpholino
nucleosides as novel inhibitors of PARP1 and PARP2: experimental
and molecular modeling analyses of their selectivity and mechanism
of action. Int J Mol Sci. 2024;25(23):12526. doi 10.3390/
ijms252312526

Chowdhuri S.P., Das B.B. Top1-PARP1 association and beyond: from
DNA topology to break repair. NAR Cancer. 2021;3(1):zcab003. doi
10.1093/narcan/zcab003

Comeaux E.Q., van Waardenburg R.C. Tyrosyl-DNA phosphodiesterase
I resolves both naturally and chemically induced DNA adducts
and its potential as a therapeutic target. Drug Metab Rev.
2014;46(4):494-507. doi 10.3109/03602532.2014.971957

Conda-Sheridan M., Reddy P.V.N., Morrell A., Cobb B.T., Marchand C.,
Agama K., Chergui A., Renaud A., Stephen A.G., Bindu L.K., Pommier
Y., Cushman M. Synthesis and biological evaluation of indenoisoquinolines
that inhibit both tyrosyl-DNA phosphodiesterase I
(Tdp1) and topoisomerase I (Top1). J Med Chem. 2013;56(1):182-
200. doi 10.1021/jm3014458

Curtin N.J., Szabo C. Poly(ADP-ribose) polymerase inhibition: past,
present and future. Nat Rev Drug Discov. 2020;19(10):711-736. doi
10.1038/s41573-020-0076-6

Das B.B., Huang S.N., Murai J., Rehman I., Amé J.-C., Sengupta S.,
Das S.K., Majumdar P., Zhang H., Biard D., Majumder H.K., Schreiber
V., Pommier Y. PARP1-TDP1 coupling for the repair of topoisomerase
I-induced DNA damage. Nucleic Acids Res. 2014;42(7):
4435-4449. doi 10.1093/nar/gku088

Dyrkheeva N., Anarbaev R., Lebedeva N., Kuprushkin M., Kuznetsova
A., Kuznetsov N., Rechkunova N., Lavrik O. Human tyrosyl-
DNA phosphodiesterase 1 possesses transphosphooligonucleotidation
activity with primary alcohols. Front Cell Dev Biol. 2020;8:
604732. doi 10.3389/fcell.2020.604732

Dyrkheeva N.S., Filimonov A.S., Luzina O.A., Orlova K.A., Chernyshova
I.A., Kornienko T.E., Malakhova A.A., … Burakova E.A.,
Stetsenko D.A., Zakian S.M., Salakhutdinov N.F., Lavrik O.I. New
hybrid compounds combining fragments of usnic acid and thioether
are inhibitors of human enzymes TDP1, TDP2 and PARP1. Int J Mol
Sci. 2021;22(21):11336. doi 10.3390/ijms222111336

Eastman P., Galvelis R., Peláez R.P., Abreu C.R.A., Farr S.E., Gallicchio
E., Gorenko A., … Wang Y., Zhang I., Chodera J.D., De Fabritiis
G., Markland T.E. OpenMM 8: molecular dynamics simulation
with machine learning potentials. J Phys Chem B. 2024;128(1):109-
116. doi 10.1021/acs.jpcb.3c06662

Elsayed W., El-Shafie L., Hassan M.K., Farag M.A., El-Khamisy S.F.
Isoeugenol is a selective potentiator of camptothecin cytotoxicity
in vertebrate cells lacking TDP1. Sci Rep. 2016;6(1):26626. doi
10.1038/srep26626

Fam H.K., Walton C., Mitra S.A., Chowdhury M., Osborne N.,
Choi K., Sun G., … Aparicio S., Triche T.J., Bond M., Pallen C.J.,
Boerkoel C.F. TDP1 and PARP1 deficiency are cytotoxic to rhabdomyosarcoma
cells. Mol Cancer Res. 2013;11(10):1179-1192. doi
10.1158/1541-7786.mcr-12-0575

Flörkemeier I., Hillmann J.S., Weimer J.P., Hildebrandt J., Hedemann
N., Rogmans C., Dempfle A., Arnold N., Clement B., Bauerschlag
D.O. Combined PARP and dual topoisomerase inhibition
potentiates genome instability and cell death in ovarian cancer. Int J
Mol Sci. 2022;23(18):10503. doi 10.3390/ijms231810503

Hoch N.C., Polo L.M. ADP-ribosylation: from molecular mechanisms
to human disease. Genet Mol Biol. 2019;43(Suppl.1):e20190075.
doi 10.1590/1678-4685-GMB-2019-0075

Hu D.-X., Tang W.-L., Zhang Y., Yang H., Wang W., Agama K., Pommier
Y., An L.-K. Synthesis of methoxy-, methylenedioxy-, hydroxy-,
and halo-substituted benzophenanthridinone derivatives as
DNA topoisomerase IB (TOP1) and tyrosyl-DNA phosphodiesterase
1 (TDP1) inhibitors and their biological activity for drug-resistant
cancer. J Med Chem. 2021;64(11):7617-7629. doi 10.1021/acs.
jmedchem.1c00318

Hu D.-X., Tang W.-L., Zhang Y., Yang H., Wang W., Agama K., Pommier
Y., An L.-K. Synthesis of methoxy-, methylenedioxy-, hydroxy-,
and halo-substituted benzophenanthridinone derivatives as
DNA topoisomerase IB (TOP1) and tyrosyl-DNA phosphodiesterase
1 (TDP1) inhibitors and their biological activity for drug-resistant
cancer. J Med Chem. 2021;64(11):7617-7629. doi 10.1021/acs.
jmedchem.1c00318

Ivanov A.A., Koval V.S., Susova O.Yu., Salyanov V.I., Oleinikov V.A.,
Stomakhin A.A., Shalginskikh N.A., Kvasha M.A., Kirsanova O.V.,
Gromova E.S., Zhuze A.L. DNA specific fluorescent symmetric dimeric
bisbenzimidazoles DBP(n): the synthesis, spectral properties,
and biological activity. Bioorg Med Chem Lett. 2015;25(13):2634-
2638. doi 10.1016/j.bmcl.2015.04.087

Jing C.-B., Fu C., Prutsch N., Wang M., He S., Look A.T. Synthetic
lethal targeting of TET2-mutant hematopoietic stem and progenitor
cells (HSPCs) with TOP1-targeted drugs and PARP1 inhibitors.
Leukemia. 2020;34(11):2992-3006. doi 10.1038/s41375-020-
0927-5

Johannes J.W., Balazs A.Y.S., Barratt D., Bista M., Chuba M.D., Cosulich
S., Critchlow S.E., … Xue L., Yao T., Zhang K., Zhang A.X.,
Zheng X. Discovery of 6-Fluoro-5-{4-[(5-fluoro-2-methyl-3-oxo-
3,4-dihydroquinoxalin-6-yl)methyl]piperazin-1-yl}-N-methylpyridine-
2-carboxamide (AZD9574): a CNS-penetrant, PARP1-
selective
inhibitor. J Med Chem. 2024;67(24):21717-21728. doi
10.1021/acs.jmedchem.4c01725

Jumper J., Evans R., Pritzel A., Green T., Figurnov M., Ronneberger
O.,
Tunyasuvunakool K., … Vinyals O., Senior A.W., Kavukcuoglu
K.,
Kohli P., Hassabis D. Highly accurate protein structure prediction
with AlphaFold. Nature. 2021;596(7873):583-589. doi 10.1038/
s41586-021-03819-2

Jurrus E., Engel D., Star K., Monson K., Brandi J., Felberg L.E.,
Brookes D.H., … Krasny R., Wei G., Holst M.J., McCammon J.A.,
Baker N.A. Improvements to the APBS biomolecular solvation software
suite. Protein Sci. 2018;27(1):112-128. doi 10.1002/pro.3280

Kawale A.S., Povirk L.F. Tyrosyl-DNA phosphodiesterases: rescuing
the genome from the risks of relaxation. Nucleic Acids Res. 2018;
46(2):520-537. doi 10.1093/nar/gkx1219

Kim D.-S., Camacho C.V., Kraus W.L. Alternate therapeutic pathways
for PARP inhibitors and potential mechanisms of resistance.
Exp Mol Med. 2021;53(1):42-51. doi 10.1038/s12276-021-
00557-3

Kim J.W., Min A., Im S.-A., Jang H., Kim Y.J., Kim H.-J., Lee K.-H.,
Kim T.-Y., Lee K.W., Oh D.-Y., Kim J.-H., Bang Y.-J. TDP1 and
TOP1 modulation in olaparib-resistant cancer determines the efficacy
of subsequent chemotherapy. Cancers. 2020;12(2):334. doi
10.3390/cancers12020334

Kornienko T.E., Chepanova A.A., Zakharenko A.L., Filimonov A.S.,
Luzina O.A., Dyrkheeva N.S., Nikolin V.P., Popova N.A., Salakhutdinov
N.F., Lavrik O.I. Enhancement of the antitumor and antimetastatic
effect of topotecan and normalization of blood counts in mice
with Lewis carcinoma by Tdp1 inhibitors – new usnic acid derivatives.
Int J Mol Sci. 2024;25(2):1210. doi 10.3390/ijms25021210

Lavrik O.I. PARPs’ impact on base excision DNA repair. DNA Repair.
2020;93:102911. doi 10.1016/j.dnarep.2020.102911Lebedeva N.A., Anarbaev R.O., Sukhanova M., Vasil’eva I.A., Rechkunova
N.I., Lavrik O.I. Poly(ADP-ribose)polymerase 1 stimulates
the AP-site cleavage activity of tyrosyl-DNA phosphodiesterase 1.
Biosci Rep. 2015;35(4):e00230. doi 10.1042/BSR20140192

Marchand C., Abdelmalak M., Kankanala J., Huang S.-Y., Kiselev E.,
Fesen K., Kurahashi K., Sasanuma H., Takeda S., Aihara H.,
Wang Z., Pommier Y. Deazaflavin inhibitors of tyrosyl-DNA phosphodiesterase
2 (TDP2) specific for the human enzyme and active
against cellular TDP2. ACS Chem Biol. 2016;11(7):1925-1933. doi
10.1021/acschembio.5b01047

Matsuno Y., Hyodo M., Fujimori H., Shimizu A., Yoshioka K. Sensitization
of cancer cells to radiation and topoisomerase I inhibitor
camptothecin using inhibitors of PARP and other signaling molecules.
Cancers. 2018;10(10):364. doi 10.3390/cancers10100364

Mirdita M., Schütze K., Moriwaki Y., Heo L., Ovchinnikov S.,
Steinegger M. ColabFold: making protein folding accessible to
all. Nat Methods. 2022;19(6):679-682. doi 10.1038/s41592-022-
01488-1

Moor N.A., Vasil’eva I.A., Anarbaev R.O., Antson A.A., Lavrik O.I.
Quantitative characterization of protein-protein complexes involved
in base excision DNA repair. Nucleic Acids Res. 2015;43(12):6009-
6022. doi 10.1093/nar/gkv569

Moretti R., Bender B.J., Allison B., Meiler J. Rosetta and the design of
ligand binding sites. In: Stoddard B. (Ed.) Computational Design of
Ligand Binding Proteins. Methods in Molecular Biology. Vol. 1414.
New York: Humana Press, 2016;47-62. doi 10.1007/978-1-4939-
3569-7_4

Murai J., Marchand C., Shahane S.A., Sun H., Huang R., Zhang Y.,
Chergui A., Ji J., Doroshow J.H., Jadhav A., Takeda S., Xia M.,
Pommier Y. Identification of novel PARP inhibitors using a cellbased
TDP1 inhibitory assay in a quantitative high-throughput
screening platform. DNA Repair. 2014;21:177-182. doi 10.1016/
j.dnarep.2014.03.006

Neudachina L., Lakiza N. Physico-Chemical Principles of the Use of
Coordination Compounds. Ekaterinburg, 2014 (in Russian)

Nguyen T.X., Abdelmalak M., Marchand C., Agama K., Pommier Y.,
Cushman M. Synthesis and biological evaluation of nitrated 7-, 8-,
9-, and 10-hydroxyindenoisoquinolines as potential dual topoisomerase
I (Top1)–tyrosyl-DNA phosphodiesterase I (TDP1) inhibitors.
J Med Chem. 2015;58(7):3188-3208. doi 10.1021/acs.jmed
chem.5b00136

O’Boyle N.M., Banck M., James C.A., Morley C., Vandermeersch T.,
Hutchison G.R. Open Babel: an open chemical toolbox. J Cheminform.
2011;3(1):33. doi 10.1186/1758-2946-3-33

Pettersen E.F., Goddard T.D., Huang C.C., Couch G.S., Greenblatt
D.M., Meng E.C., Ferrin T.E. UCSF Chimera – a visualization
system for exploratory research and analysis. J Comput Chem.
2004;25(13):1605-1612. doi 10.1002/jcc.20084

Pommier Y., Leo E., Zhang H., Marchand C. DNA topoisomerases and
their poisoning by anticancer and antibacterial drugs. Chem Biol.
2010;17(5):421-433. doi 10.1016/j.chembiol.2010.04.012

Pommier Y., Huang S.N., Gao R., Das B.B., Murai J., Marchand C.
Tyrosyl-DNA-phosphodiesterases (TDP1 and TDP2). DNA Repair.
2014;19:114-129. doi 10.1016/j.dnarep.2014.03.020

Quiroga R., Villarreal M.A. Vinardo: a scoring function based on autodock
vina improves scoring, docking, and virtual screening. PLoS
One. 2016;11(5):e0155183. doi 10.1371/journal.pone.0155183

Ray Chaudhuri A., Nussenzweig A. The multifaceted roles of PARP1
in DNA repair and chromatin remodelling. Nat Rev Mol Cell Biol.
2017;18(10):610-621. doi 10.1038/nrm.2017.53

Salentin S., Schreiber S., Haupt V.J., Adasme M.F., Schroeder M. PLIP:
fully automated protein–ligand interaction profiler. Nucleic Acids
Res. 2015;43(W1):W443-W447. doi 10.1093/nar/gkv315

Schreiber V., Dantzer F., Ame J.-C., de Murcia G. Poly(ADP-ribose):
novel functions for an old molecule. Nat Rev Mol Cell Biol. 2006;
7(7):517-528. doi 10.1038/nrm1963

Sherstyuk Y.V., Ivanisenko N.V., Zakharenko A.L., Sukhanova M.V.,
Peshkov R.Y., Eltsov I.V., Kutuzov M.M., Kurgina T.A., Belousova
E.A., Ivanisenko V.A., Lavrik O.I., Silnikov V.N., Abramova T.V.
Design, synthesis and molecular modeling study of conjugates of
ADP and morpholino nucleosides as a novel class of inhibitors of
PARP-1, PARP-2 and PARP-3. Int J Mol Sci. 2019;21(1):214. doi
10.3390/ijms21010214

Smith L.M., Willmore E., Austin C.A., Curtin N.J. The novel poly(ADPribose)
polymerase inhibitor, AG14361, sensitizes cells to topoisomerase
I poisons by increasing the persistence of DNA strand
breaks. Clin Cancer Res. 2005;11(23):8449-8457. doi 10.1158/1078-
0432.ccr-05-1224Sukhanova M.V., Khodyreva S.N., Lavrik O.I. Poly(ADP-ribose) polymerase-
1 inhibits strand-displacement synthesis of DNA catalyzed
by DNA polymerase β. Biochemistry (Moscow). 2004;69(5):558-
568. doi 10.1023/b:biry.0000029855.68502.fa

Susova О.Y., Kаrshieva S.S., Kostyukov А.А., Мoiseevа N.I., Zaytseva
Е.А., Kаlabina K.V., Zusinaite Е., Gildemann K., Smirnov N.М.,
Аrutyunyan А.F., Zhuze А.L. Dimeric bis-benzimidazole-pyrroles
DB2Py(n) – AT-site-specific ligands: synthesis, physicochemical
analysis, and biological activity. Acta Naturae. 2024;16(1):86-100.
doi 10.32607/actanaturae.27327Szanto M., Yelamos J., Bai P. Specific and shared biological functions
of PARP2 – is PARP2 really a lil' brother of PARP1? Expert Rev
Mol Med. 2024;26:e13. doi 10.1017/erm.2024.14

Wang P., Elsayed M.S.A., Plescia C.B., Ravji A., Redon C.E., Kiselev
E., Marchand C., Zeleznik O., Agama K., Pommier Y., Cushman
M. Synthesis and biological evaluation of the first triple
inhibitors of human topoisomerase 1, tyrosyl-DNA phosphodiesterase
1 (Tdp1), and tyrosyl-DNA phosphodiesterase 2 (Tdp2).
J Med Chem. 2017;60(8):3275-3288. doi 10.1021/acs.jmedchem.
6b01565

Wang R., Lai L., Wang S. Further development and validation of empirical
scoring functions for structure-based binding affinity prediction.
J Comput Aided Mol Des. 2002;16(1):11-26. doi 10.1023/a:
1016357811882

Yang H., Zhu X.-Q., Wang W., Chen Y., Hu Z., Zhang Y., Hu D.-X.,
Yu L.-M., Agama K., Pommier Y., An L.-K. The synthesis of furoquinolinedione
and isoxazoloquinolinedione derivatives as selective
Tyrosyl-DNA phosphodiesterase 2 (TDP2) inhibitors. Bioorg Chem.
2021;111:104881. doi 10.1016/j.bioorg.2021.104881

Yang H., Qin C., Wu M., Wang F., Wang W., Agama K., Pommier Y.,
Hu D., An L. Synthesis and biological activities of 11‐ and 12‐substituted
benzophenanthridinone derivatives as DNA topoisomerase IB
and tyrosyl‐DNA phosphodiesterase 1 inhibitors. ChemMedChem.
2023;18(10):e202200593. doi 10.1002/cmdc.202200593

Zakharenko A., Khomenko T., Zhukova S., Koval O., Zakharova
O., Anarbaev R., Lebedeva N., Korchagina D., Komarova N.,
Vasiliev V., Reynisson J., Volcho K., Salakhutdinov N., Lavrik O.
Synthesis and biological evaluation of novel tyrosyl-DNA phosphodiesterase
1 inhibitors with a benzopentathiepine moiety.
Bioorg Med Chem. 2015;23(9):2044-2052. doi 10.1016/j.bmc.
2015.03.020

Zakharenko A.L., Luzina O.A., Chepanova A.A., Dyrkheeva N.S.,
Salakhutdinov N.F., Lavrik O.I. Natural products and their derivatives
as inhibitors of the DNA repair enzyme tyrosyl-DNA phosphodiesterase 1. Int J Mol Sci. 2023;24(6):5781. doi 10.3390/ijms
24065781

Zeng Z., Sharma A., Ju L., Murai J., Umans L., Vermeire L., Pommier
Y.,
Takeda S., Huylebroeck D., Caldecott K.W., El-Khamisy S.F. TDP2
promotes repair of topoisomerase I-mediated DNA damage in the
absence of TDP1. Nucleic Acids Res. 2012;40(17):8371-8380. doi
10.1093/nar/gks622

Zhang M., Wang Z., Su Y., Yan W., Ouyang Y., Fan Y., Huang Y.,
Yang H. TDP1 represents a promising therapeutic target for overcoming
tumor resistance to chemotherapeutic agents: progress and
potential. Bioorg Chem. 2025;154:108072. doi 10.1016/j.bioorg.
2024.108072

Zhang X.-R., Wang H.-W., Tang W.-L., Zhang Y., Yang H., Hu D.-X.,
Ravji A., Marchand C., Kiselev E., Ofori-Atta K., Agama K., Pommier
Y., An L.-K. Discovery, synthesis, and evaluation of oxynitidine
derivatives as dual inhibitors of DNA topoisomerase IB (TOP1)
and tyrosyl-DNA phosphodiesterase 1 (TDP1), and potential antitumor
agents. J Med Chem. 2018;61(22):9908-9930. doi 10.1021/
acs.jmedchem.8b00639

Zhang Y., He X., Yang H., Liu H., An L. Robustadial A and B from
Eucalyptus globulus Labill. and their anticancer activity as selective
tyrosyl‐DNA phosphodiesterase 2 inhibitors. Phytotherapy Res.
2021;35(9):5282-5289. doi 10.1002/ptr.7207

